# Caregiver identity in care partners of persons living with mild cognitive impairment

**DOI:** 10.1177/1471301221994317

**Published:** 2021-02-17

**Authors:** Brooke E Beatie, Corey S Mackenzie, Laura Funk, Dylan Davidson, Lesley Koven, Kristin A Reynolds

**Affiliations:** Department of Psychology, 194379 University of Manitoba, Winnipeg, Manitoba, Canada; Department of Sociology and Criminology, 8664 University of Manitoba, Winnipeg, Manitoba, Canada; Department of Psychology, 12359 University of Manitoba, Winnipeg, Manitoba, Canada; Department of Clinical Health Psychology, 12359 University of Manitoba, Winnipeg, Manitoba, Canada; Department of Psychology, 12359 University of Manitoba, Winnipeg, Manitoba, Canada

**Keywords:** informal caregiving, caregiver stress, memory loss, older adults, caregiver support, mild cognitive impairment

## Abstract

Research on caregiver identity in the context of memory impairment has focused primarily on more advanced stages of the cognitive impairment trajectory (e.g., dementia caregivers), failing to capture the complex dynamics of early caregiver identity development (e.g., MCI; mild cognitive impairment caregivers). The aim of this study was to develop a nuanced understanding of how caregiver identity develops in family and friends of persons living with MCI. Using constructivist grounded theory (ConGT), this study explored caregiver identity development from 18 in-depth interviews with spouses (*n* = 13), children (*n* = 3), and friends (*n* = 2) of persons recently diagnosed with MCI. The overarching themes influencing MCI caregiver identity development included MCI changes, care-related experiences, “caregiver” interpretation, and approach/avoidance coping. These themes influenced how participants primarily identified, represented as *I am a caregiver, I am not a caregiver, *or* liminality* (i.e., between their previous identity and a caregiver identity). Irrespective of their current self-identification, all conveyed thinking about their “future self,” as providing more intensive care. MCI caregiver identity development in family and friends is a fluid and evolving process. Nearly all participants had taken on care tasks, yet the majority of these individuals did not clearly identify as caregivers. Irrespective of how participants identified, they were engaging in care, and would likely benefit from support with navigating these changes and their new, ambiguous, and evolving roles.

Literature on caregiver identity in the context of aging and cognitive impairment has tended to focus on caregiving for persons living with dementia (Cross et al., 2018). However, there is growing interest in the experiences of people with mild cognitive impairment (MCI) and the family and friends who are supporting them (Joosten-Weyn Banningh et al., 2013; Morris et al., 2020; Petersen et al., 2011; Pine, 2018; Roberto et al., 2011). MCI is characterized by deterioration in memory, attention, and cognitive function beyond what is expected based on age and education. It is commonly viewed as a transitional phase between normal cognitive aging and dementia (Petersen et al., 2014). For example, roughly 10–15% of people diagnosed with MCI develop dementia within the first year, increasing to 80%–90% after approximately 6 years (Eshkoor et al., 2015). However, not everyone diagnosed with MCI develops dementia, and people with this condition vary greatly in terms of the presence or absence of memory impairment, the number of impaired cognitive domains, and symptom severity (Prince et al., 2013). Cognitive impairment(s) must be noticeable, but not severe enough to significantly impact daily functioning (Petersen et al., 2014). Understandably, this diagnosis is associated with variability and ambiguity both for those diagnosed (Gomersall et al., 2015) and their care partners.

Research on MCI care partners concludes that they too are a heterogeneous group, performing a variety of tasks that are largely dependent on the degree of impairment of the person with MCI (Seeher et al., 2013). Although different labels are used when referring to individuals in this group (e.g., family caregiver, informal caregiver, and caregiver role), for clarity and consistency within the recent literature (Joosten-Weyn Banningh et al., 2013; Morris et al., 2020; Pine, 2018; Roberto et al., 2011), we will refer to this group as “care partners.” Despite diversity in responsibilities faced by MCI care partners, they commonly assume a number of caregiving tasks (Fisher et al., 2011; McIlvane et al., 2008), which can sometimes lead to stress, burden, and negative effects on mental and cognitive health (Blieszner & Roberto, 2010). Yet, while this group could benefit from psychosocial support services (Ryan et al., 2010), they are unlikely to use them (Eifert et al., 2015). Although several barriers to accessing such services have been identified (e.g., insufficient availability of services, lack of perceived need for help, lack of service knowledge, and financial concerns; Morgan et al., 2002; Peterson et al., 2016), a key reason is that many of these individuals do not self-identify as caregivers (Eifert et al., 2015). To best support MCI care partners, researchers must take a step back to examine what it means to be an “MCI caregiver.”

Caregiver identity is distinct from caregiving behavior **(**Funk, 2019). Although MCI care partners help with tasks (e.g., managing finances and appointments, and ensuring medication adherence; Fisher et al., 2011; McIlvane et al., 2008; Woolmore-Goodwin et al., 2016), how they identify is significantly less clear. Two useful theories contribute to our understanding of caregiver identity development: Positioning Theory (Harré & van Langenhove, 1999; O’Connor, 2007) and Caregiver Identity Theory (CIT; Montgomery & Kosloski, 2009). These theories align in suggesting that (1) the caregiver experience is individualized, (2) caregivers differ in the degree of tension or distress around assuming a caregiver role or identity, and (3) caregiver identity is influenced by and emerges from a preexisting relationship. However, CIT applies a developmental approach to identity development, whereas positioning theory suggests it is a more variable and fluid process. Further literature on caregiver identity suggests there is great variability in how and when people self-identify. For example, people may anticipate caregiving roles before ever beginning to provide such care (Sörensen, 1998). A caregiver identity may also continue even after an individual is no longer providing care (e.g., bereaved caregivers becoming advocates for other caregivers; Larkin, 2009). Interestingly, some individuals may provide care without ever self-identifying as a caregiver, which suggests a sense of discomfort with this identity (Funk, 2019; Hendersen, 2001; O’Connor, 1999). Overall, theory and prior research provide a solid foundation for exploring caregiver identity early in the caregiving process.

There is a noticeable gap in the literature aimed at understanding caregiver identity development in MCI. In fact, MCI care partners may not necessarily identify as caregivers, despite being labeled as such in studies (Dean et al., 2014; Morris et al., 2020; Paradise et al., 2015; Seeher et al., 2013). This may be especially true for MCI care partners for two reasons. First, because MCI does not cause impairment in daily functioning (Petersen et al., 2014), care partners may not view the type of care they provide as significant enough to elicit an identity shift. Second, the expression of impairment in people with MCI varies (Roberto et al., 2013), which may create ambivalence in care partners regarding whether they fulfill or identify with a caregiver role versus their preexisting relational role (e.g., spouse).

We are aware of only one previous study addressing identity development within the context of MCI (Woolmore-Goodwin et al., 2016). Care partners symbolically wore “multiple situational masks” to cope with accumulated progressive losses as they adjusted to their new and evolving identities. They reported “forbidden thoughts” (e.g., “sometimes I don’t want to be there for her”)—possible by-products of struggles accepting the social expectations involved in the new role. The authors highlighted the need to further explore the extent to which MCI caregivers modify their self-concepts and identities. This aligns with previous research (Adams, 2006; Morris et al., 2020) which suggests that a deeper understanding of the early stages of caregiver identity development could provide insight into the attributes of persons who may be more prone to negative mental health outcomes and burnout. To address this gap, the current study examines how caregiver identity develops in care partners of people living with MCI. Insights into caregiver identity development in the context of MCI can inform the delivery of support services tailored to MCI care partners.

## Methods

### Research design

This study was guided by constructivist grounded theory (ConGT) methodology (Charmaz, 2014). Constructivist grounded theory methodology is informed by the notion that knowledge is co-constructed through interactions with others and through historical and cultural norms that shape individuals’ lives to produce an interpretable understanding of the subjective meaning (Creswell, 2014). This methodological approach is suited to exploring understudied and complex social processes while generating theoretical insights with practical applications (Charmaz, 2014).

### Study setting

We recruited 18 participants from a memory clinic in a large health sciences center of a major Canadian city. The memory clinic provides assessment and memory group intervention for older adults with cognitive impairment (including MCI and dementia) and their “program partners.” Program partners are spouses, children, or friends that patients choose to bring with them to their initial neuropsychological assessment appointment.

### Sampling and participants

Consistent with grounded theory methodology, we used theoretical sampling to select participants according to emergent findings from ongoing data collection and subsequent analyses (Charmaz, 2014). Between February 2018 and November 2019, a geropsychologist approached 24 program partners to inform them of the study, and 18 agreed to participate (response rate of 75%). Program partners were eligible to enroll if (a) their family member or friend received an MCI diagnosis within the past six months, and (b) they were fluent in English and had no obvious signs of cognitive impairment as determined by the geropsychologist. As themes began to emerge, additional participants were selected to enrich our theoretical understanding of caregiver identity development. Our research team (six members with professional and research backgrounds in geropsychology, clinical psychology, and sociology) continued to examine emerging themes and adjusted the interview protocol as needed. We continued to recruit participants until the data reached theoretical sufficiency, where few new ideas were emerging with additional interviews (Dey, 1999).

### Data collection

Consenting participants completed a semi**-**structured, approximately 75-min interview with the first author in a location of their choice (e.g., their home or memory clinic). The interview protocol (see Supplementary Material) began with the central, open-ended question, “Can you start by telling me about your relationship with [care-recipient diagnosed with MCI]?” Depending on the response to this question, additional questions explored: details about their relationship before their friend/family member showed signs of MCI, societal and cultural norms (e.g., feelings of responsibility to provide care), contextual changes (e.g., the person with MCI’s symptoms), changes in their own behavior (e.g., care tasks), and how they view themselves since their friend/family member’s decline. In line with ConGT, our interview questions evolved between interviews so as to build upon and generate theories through successive levels of data analysis and conceptual development. The first author documented field notes after each interview, which also helped identify preliminary themes and guide subsequent interviews. Interviews were audio-recorded, de-identified, and professionally transcribed.

### Data analysis

Consistent with ConGT (Charmaz, 2014), the interviewer, research team, and participants mutually co-constructed meaning regarding MCI caregiver identity development during data collection and analysis; the resulting theoretical model is an interpretation of this phenomenon. We used a constant comparative approach to analyze the data through three stages: initial coding, focused coding, and theoretical coding (Charmaz, 2014).

Transcripts, memos, and field notes were read, reread, and two authors conducted line-by-line “initial coding” of the transcripts, which focused on preliminary concepts. Codes remained close to the data, with a particular focus on participants’ use of language to generate codes as often as possible (e.g., “is this memory problems or is this the depression,” “I am more of a teacher and a mother than I am as a wife,” “caregiving is a lot of responsibility…we’re not there yet”). Our evolving list of initial codes, along with a selection of six transcripts, was reviewed and discussed with three additional authors.

As additional interviews were conducted, each new transcript was coded and compared to previously analyzed interviews, which helped refine existing codes and generate new codes that were more conceptual and described larger amounts of data (e.g., “my future self” code). The first author developed diagrams to further conceptualize and organize the initial and focused codes, which also provided theoretical direction during the analytic process.

Finally, during theoretical coding, our team compared, refined, and related focused codes to each other, to develop main themes and subthemes for the theoretical model. Consistent with ConGT, our team transitioned back and forth between these three phases of coding. We continued to compare incoming data with analyzed data to ensure we had a thorough understanding of participants’ caregiver identity development, resulting in the final theoretical model.

### Rigor

Throughout the analytic process, we maintained a rigorous audit trail, including coded interview transcripts and field notes, written case summaries for each participant by two authors, diagramming excerpts, and ongoing memos from the team to enhance analytic credibility and originality. Consolidated memos throughout the entire research process provided a visual trail of the various research stages, from formulating the research question to data collection and theoretical development. Additionally, given that under the constructivist paradigm, data collection and analyses are influenced by interactions between the researcher and participants (in addition to social, cultural, and structural contexts; Charmaz, 2014), the first author documented her personal reflections and how their background (e.g., their experiences as a clinical psychology doctoral student and clinician, and personal familial experience with caregiving) and presence affected their interactions with participants. Throughout the data collection and analytic process, these considerations were discussed with the research team and guided future participant interactions and analysis. Finally, to enhance the credibility and consistency of our findings with regard to the research participants and context, debriefing and member-checking (Charmaz, 2014) were conducted after each interview. Due to the rigorous analytic process, the constructed theoretical model offers a novel conceptual interpretation of MCI caregiver identity development.

## Findings

### Sample description

Participants were spouses (*n* = 13), children (*n* = 3), and friends (*n* = 2) of persons recently diagnosed with MCI, including amnestic (*n* = 16) and non-amnestic (*n* = 2) MCI. The majority of participants (72%) lived with the person with MCI. Their ages ranged from 39 to 89 years (M = 69.4, SD = 12.8), 61% identified as female, and 94% as white (one identified as Indigenous). Additional information on demographic characteristics is presented in Table 1.

**Table 1.** Participant demographics.

Characteristics		*n*	*%*
Relationship to partner with MCI			
Spouse		13	72
Child		3	17
Friend		2	11
Living with partner with MCI			
Yes		13	72
No		5	28
Partner’s MCI subtype			
Amnestic		16	895
Non-amnestic		2	11
Gender			
Male		7	39
Female		11	61
Ethnicity			
White		17	94
Indigenous		1	6
Marital status			
Married/common law		16	89
Widowed		2	11
Highest education completed			
Grade 12		7	39
Undergraduate degree		7	39
Graduate degree		4	22
Occupational status			
Retired		15	83
Part-time		1	6
Full-time		2	11
Estimated annual household income			
Under US$25,000		2	11
US$25,001–US$50,000		5	28
US$50,001–US$75,000		4	22
Over US$75,001		7	39
	*Range*	*M*	*SD.*
Age	39–89	69.4	12.8

*N* = 18. MCI: Mild cognitive impairment.

### MCI caregiver identity development

**Figure 1.**
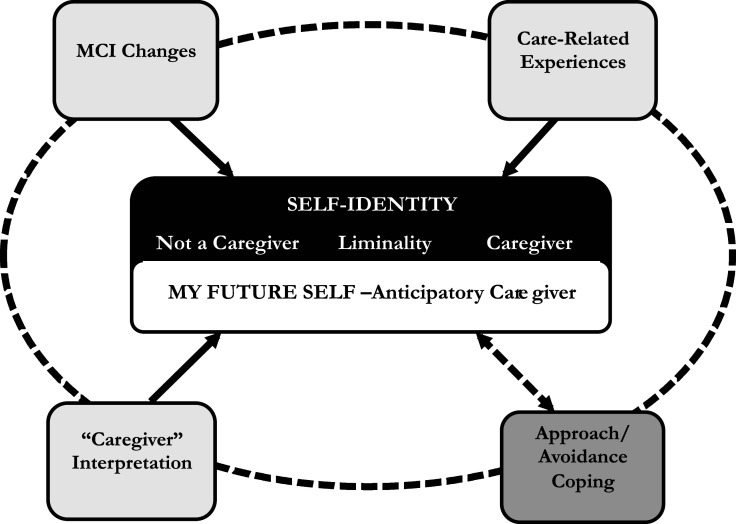
Mild cognitive impairment caregiver identity development thematic model.

Our analysis generated six major thematic categories (Figure 1) representing influences on MCI identity development: (1) MCI changes, (2) care-related experiences, (3) “caregiver” interpretations, and (4) approach/avoidance coping. These themes influenced how participants (5) currently self-identified, which were refined to: *I am a caregiver, I am not a caregiver*, and *liminality*. Related to participants’ self-identities were their reflections about how they envisioned their (6) “future self,” as an anticipatory caregiver.

### MCI changes

#### MCI is confusing, unpredictable, and sometimes transient

Participants’ descriptions of the cognitive symptoms of their family member or friend varied, consistent with the ambiguous and heterogeneous nature of MCI. Most frequently reported were memory problems that were not “extremely debilitating.” Participants reflected that they were sometimes confused by observed changes, which were unpredictable and transient. Complicating matters further, some participants noticed co-occurring health problems (e.g., depression, anxiety, and hearing difficulty), which “muddied the waters” in terms of how to understand or make sense of what was happening. Many participants discussed challenges trying to understand, “Is this the MCI, or is this something else?” As this husband explained:

*“She doesn’t only have a bit of memory issues but she also suffers from depression. And they sort of feed off each other as far as that goes…and it comes and goes. She still has some really good times where she’s sharp as a tack, remembers pretty well everything. And then there’s other situations which, you know, you can talk to her, and 10 minutes later she’ll ask you the same question as though it’s never been talked about.”*

#### Shifts and imbalance in roles creates tension

Participants reflected on how their roles began shifting as they observed MCI symptoms emerging, and in turn, influenced how they viewed their roles and identities. Many described feeling as though they were no longer on “equal terrain” with their family member/friend, an experiential shift from the norm of their preexisting relationship roles, which contributed to the tension. They described sometimes feeling frustrated, resentful, and irritable toward their family member/friend and the MCI, often followed by guilt about these emotions. Some participants described resisting this “role shift” because they did not want the diagnosis (and what it represents) to be true. They struggled with accepting what was happening to their family member:

*“So I sort of feel sometimes like I was kind of assuming this parent role almost with him…which I don’t want to be. I’m not a parent right now, so don’t want to be nagging my dad. And I have to be careful too because I don’t want to… (participant became tearful). I get frustrated with him sometimes, and I sort of have to take a step back and just say, you know, I feel a little bit guilty about that almost.”*

### Care-related experiences

In addition to MCI-related changes, caregiver identity appeared to be embedded within and influenced by two broader types of care-related experiences.

#### Past experiences with caregiving

Participants’ prior histories and life courses appeared to shape their “caregiver” self-identification. They recollected how social norms, cultural norms, gendered care expectations, and familial roles (e.g., providing care for ailing family members earlier in life as a teen/young adult) influenced their current self-concepts. Previous caregiving experiences appeared to be closely tied to current interpretations of the meaning of “caregiver,” as discussed by this wife:

*“It’s just my life experiences. When I was young I was about 15 years old I looked after a woman with arthritis for two weeks one summer while her family went away on holidays. Like I started the caregiving early. It’s inbred… and I think it was, I’ve always wanted to be a nurse, so I think that I was always like that.”*

#### Social network influences

Another contextual factor shaping caregiver identity was the influence of participants’ social networks, including how participants were treated (i.e., symbolically viewed) by the healthcare system and other informal network members. Some participants reported direct feedback, as when the caregiver label was given to them by other family or healthcare practitioners, who would refer to them as the “caregiver.” Additionally, many participants were indirectly influenced by their social network—hearing their friends or family discuss personal experiences with dementia. In turn, participants compared their experience with MCI to others’ experiences with dementia, which shaped how they were currently viewing their role, and how their role could be affected in the future, as this husband illustrated:

*“And her [wife with MCI] father has dementia. I mean he’s now in a locked-down situation in a home. His wife was having trouble controlling him, and we’ve heard stories and watched it. And so I’m not sure whether that’s been sort of an education if you want to call it that. But it has [been] for me because I’m looking at this from afar. And I’m hoping our relationship doesn’t go there.”*

### “Caregiver” interpretation

Participants’ interpretations of the meaning of being a caregiver shaped their self-identities and their thoughts about their future caregiving self. Four types of interpretations emerged from our data.

#### Caregiving is a power imbalance

The majority of participants interpreted caregiving as signifying an observable or emotionally felt imbalance. Some noted that caregiving involves more intensive behavioral tasks or responsibilities, beyond the norm for their role as a partner, child, or friend. Participants noted that these additional responsibilities create a power imbalance, which would not occur from “normal” aging. As this wife explains:

*“I think a partner you share and you talk about things and decide who’s going to do what. A caregiver to me is in control. Makes the decisions. Tells you about big jobs, you know.”*

#### Role ambivalence

Many participants conveyed confusion and uncertainty regarding what it means to be a caregiver. They voiced uncertainty about whether their new additional responsibilities were part of their previous roles (e.g., questioning whether being a spouse and a caregiver was in fact equivalent, or not). Expected norms were not always clear, as this husband described when asked about the difference between being a husband and caregiver:

*“I guess there’s certain things that have changed in our life that way. Just being the two of us, if there had been a subservient thing or an assertive thing, but it’s always been an equal family thing, and so I can’t really make any other distinction of it. It seems to be humming along really nicely until she forgets something that’s all… I can’t really describe it. I don’t know. I guess it’s from too many years of being together. I can’t describe any difference.”*

#### Emphasis on medical/professional definition

Some participants emphasized a professional or medical definition, describing caregiving as a professional job (i.e., synonymous with nursing or other healthcare work) with a clear, formalized set of tasks or responsibilities. For some participants, invoking this definition may be an active attempt to resist a caregiver identity, since this definition provides a stark contrast to their own self-identity. This husband, who did not self-identify as a caregiver, explained:

*“There is an impersonality that a caregiver provides; it’s a servant if I can say that. The 21* ^*st*^ *century, we don’t have servants anymore. But when you think back and look at times gone by, the servants in the household would dress her and look after her. That’s the caregiver. And that’s acceptable from a caregiver. But not from your equal.”*

#### Caregiving as both burdensome and purposeful/important

Many participants interpreted caregiving with burden and strain. Asked whether he sees himself as a caregiver, this husband implicitly defines “caregiving” as something that is burdensome:

*Yea, I think it does, but if you were rating it on a percentage scale, it would be 10, 20%. It’s not super taxing to me at this point in time.”*

In contrast, others spoke of caregiving as an important, admirable, and powerful role that provided them with purpose. Occasionally, people vacillated throughout the interview, expressing both challenges and benefits of caregiving.

### Approach versus avoidance coping

Participants responded to MCI changes in ways that we categorized into *approach* and *avoidance coping.* Nearly all participants (*n* = 17) had taken on additional tasks (e.g., appointment and schedule management, reminders, and financial decisions) since the onset of their friend/family member’s symptoms. However, some participants appeared to do so in a more voluntary and active way, whereas others demonstrated a more avoidant coping style.

The large majority of participants demonstrated an “approach response” to MCI changes. They described acting as an advocate, initiating referrals or requesting doctor’s appointments, and voluntarily performing additional tasks based on a sense of responsibility. Interestingly, more active and engaged approaches did not necessarily mean that participants identified as caregivers; this is represented by the dashed line between the *self-identit*y and *approach/avoidance coping* themes in Figure 1. In contrast, a few participants described more of an “avoidant” response—not wanting to face what is happening and what it means for them, their relationship, and their lives. Not surprisingly, those participants who coped with avoidance tended not to identify as caregivers. These participants appeared closed off, minimizing the changes or their additional tasks. For example, a wife described how she was making more decisions which used to be her husband’s responsibility, but then minimized this: “It’s not like they’re big important decisions.”

### Self-identity (“Who am I” in relation to the person with MCI)

Participants were explicitly asked whether they identified as a caregiver near the end of the interview. A minority of participants self-identified as a caregiver, whereas some did not identify with this role at all. The majority of participants described existing in the “in-between”— identifying in a space of liminality.

#### I am a caregiver

Of the few participants who self-identified as caregivers, this identity appeared to supersede their other role or preexisting relational identities (e.g., wife, friend, or son). These participants illustrated a clear relational shift. They spoke of the loss of their preexisting identity, often referring to it in past tense, with finality. Their identity shift, to seeing themselves as caregivers, appeared to be primarily shaped by the severity of the MCI changes, past caregiving experiences, and interpretations of caregiving as a “power imbalance.” These participants appeared to no longer view themselves as equals with their partners. Particularly, this was observed in how they believed they were depended on in distinctly different ways than in their pre-existing relationships. As this wife explained:

*“Well, I have become a caregiver. Whereas before we were both very, although supporting each other, very independent, strong people. So that’s been a real change.”*

#### I am not a caregiver

Several participants rejected the caregiver label; their preexisting relational identities and other facets of their identities remained dominant. As one husband stated, “I’m a guy. That’s it.” Their firm stance—*not* identifying as caregivers—sometimes implied a sense of denial, rejection, and nonacceptance of the very idea of being a caregiver. In part, this appeared to be a sign of maintaining hope, attempting to avoid anxiety about impending decline and imbalance in the relationship, or maintaining boundaries around commitments and family obligations. Additionally, rejecting a caregiver identity was often coupled with efforts to preserve and protect dignity and respect for their friend or family member. For example, when exploring the term “caregiver,” many participants elaborated in detail about their family member or friend’s capabilities, independence, and special qualities. These care partners often emphasized how the current situation is not as bad as it could be with statements such as “we are not there yet.”

#### Liminality

Participants in this category conveyed a sense of ambivalence, or of being “in-between” their previous way of identifying and a newer sense of a caregiver identity, often contradicting themselves during the interview. Others described this “in-between” as a state of just beginning to see themselves as a caregiver, while not fully identifying as such. Identity liminality appeared to be influenced by the ambiguity and/or mild nature of their partner’s cognitive changes, often contrasting their partner’s MCI symptoms to more severe illnesses or impairments. Their caregiver interpretation of role ambivalence, including a sense of confusion and uncertainty with what being a caregiver means, also appeared to influence their identity liminality. When asked if she considers herself as a caregiver in relation to her father, this daughter responded:

*“I’m not sure. Like I do. I guess. I’ve sometimes (long pause) I must (long pause). In my mind, as you’re saying it, I associate caregiver with like he’s dying or like he’s really old. And I guess I don’t see him in that way yet. So I guess maybe that’s just how I associate the word as well a bit. So yes and no. Like I know I’m taking care of him. I guess I would say I’m taking care of him, I’m not his caregiver. But I know I’m doing a lot of that work so...I don’t know.”*

Noticeably, this daughter struggles to make sense of how she sees herself, vacillating between identifying and not identifying as a caregiver, yet highlighting that she is in fact taking care of her father (i.e., an approach response). It is important to note, similar to many participants identifying in liminality, adopting a caregiver identity was not a prerequisite to provide care or support. Some described their approach response as entangled as part of both their preexisting identity and beginning stages of a possible emerging caregiver identity, as noted above. In contrast, other participants in liminality felt the way they were supporting their partner was solely because of their preexisting role (e.g., “To me it is my responsibility, it’s my job as her son,”).

### Future self: The anticipatory caregiver

Irrespective of how participants currently identified, all conveyed thinking about their “future self”— most often, envisioning themselves providing more intensive care. We coded discussions of the future in the following three ways.

#### Worrying about future (uncertain) decline

When reflecting on their future self, participants often described worrying about the clinical course and prognosis of MCI, a possible dementia diagnosis, and their own decline. As one husband illustrates:

*“I really don’t want to get into that stage where she has to go somewhere, or I have to go somewhere. And if I had to go somewhere first for some physical reason or something, I would worry myself sick about her.”*

#### Anticipatory grief

Reflecting on the future, participants commonly conveyed a future sense of loss—of time and of what life was “supposed to be.” During these moments, it appeared that participants were already mourning the loss of what could have been, as depicted by this friend’s thoughts on what their future relationship might become:

*“I think of that often. Or not often, once in a while. And it makes me sad. I’m sad because I’ll lose a friend.”*

#### Maintaining hope

Last, amid anticipations of their future self as a more intensive caregiver, including worries about their own future decline and grief, were glimpses of participants’ attempts to maintain hope, for the person with MCI, their relationship, and themselves. As one husband explained:

*“I still hope there’s some kind of way of addressing that [wife’s MCI prognosis] a little better and dealing with it. I’m not sure if there’s, can’t say cure but, something changes for the better. Sometimes I just directly tell her that it’s going to get better. You know, don’t worry about it, it’s nothing to really worry about right now because we’re still moving ahead and there’s still a chance that things will turn right around in my mind. I’m trying to be very optimistic.”*

## Discussion and Implications

This study contributes to a new and growing body of literature, providing novel insights into care partners for persons living with MCI, deepening our understanding of caregiver identity development in this understudied population. Central contextual lenses that influenced how care partners’ self-identified included MCI-related changes, care-related experiences, “caregiver” interpretations, and approach/avoidance coping styles. Irrespective of how participants currently self-identified, all anticipated that their “future self” would provide more intensive care. These findings highlight the complex process of caregiver identity development in MCI care partners and have implications for improving access to supports for these individuals.

### MCI identity development: Connecting to the caregiver identity literature

Our findings suggest that MCI caregiver identity is neither binary nor fixed. Rather, it is better understood as fluid and evolving across a continuum as depicted in Figure 1. Although in some respects CIT (Montgomery & Kosloski, 2009) and positioning theory (Harré & van Langenhove, 1999; O’Connor, 2007) may be perceived as contrasting theories of caregiver identity development (i.e., developmental change versus fluid and variable process), aspects of our findings align with and provide insights relevant to both theories.

Caregiver identity theory conceptualizes caregiving as a series of developmental identity transitions that occur predominantly because of changes in the care context (e.g., onset of new symptoms; Montgomery & Kosloski, 2009). Caregiver identity theory postulates identity development as comprising five phases and suggests that during Phase 2, individuals come to view themselves as caregivers when realizing their care tasks are beginning to extend beyond the scope of their preexisting relational roles. In line with CIT, our findings highlight MCI changes as a primary theme influencing caregiver self-identification. However, our findings add to CIT in suggesting other psychosocial factors (e.g., care-related experiences, “caregiver” interpretations) that may also influence identity development.

Moreover, the majority of participants identified within liminality; the fluid and variable nature of this identity type is consistent with positioning theory. According to this theory, an identity is discursively produced, when one creates a new framework for understanding and constructing meaning about their actions (Harré & van Langenhove, 1999; O’Connor, 2007). One previous study used positioning theory to examine caregiver self-identification and found that recognizing oneself as a caregiver was constructed mainly through interactions with others (O’Connor, 2007), a finding confirmed in the present study. Indeed, we found that some participants’ caregiver identities shifted throughout interviews as they were directly asked (perhaps for the first time), thought about, and discussed whether they considered themselves caregivers. Figure 1 indicates that the fluid sense of identity was related to the other themes that emerged, such as previous life experiences and shifting MCI symptoms and relational roles, highlighting the complexity of this issue.

### Anticipatory caregiving in MCI care partners

Anticipatory caregiving was a main theme among participants, irrespective of their current self-identification. Envisioning their future selves as more intensive types of caregivers elicited discussions of possible future loss and anticipatory grief. This aligns with previous research on MCI care partners’ experience of ambiguous loss (i.e., phases of bereavement in advance of losing a significant person; Blieszner et al., 2007; Garand et al., 2012). Woolmore-Goodwin et al. (2016) found that MCI care partners’ anticipatory grief involved multiple losses (e.g., of preexisting relationship, control, and hope), which shaped how care partners viewed themselves and made them question their ability to continue to provide care in the long term (Woolmore-Goodwin et al., 2016). These worries were also common in our participants’ accounts of anticipatory caregiving, which included worries about the future and uncertain decline of their friend/family member, and how they would be able to cope with these potential changes, as is commonly reported in the MCI literature (Beard & Neary, 2013; Gomersall et al., 2015; Gomersall et al., 2017).

In addition to the loss of hope noted by Woolmore-Goodwin et al. (2016), several of our participants discussed ways they were trying to maintain hope as they navigate the MCI prognosis, and what it means for them and their relationship with their friend/family member living with MCI. The uncertain and fluctuating nature of MCI may be simultaneously comforting, an opportunity for hope for MCI care partners and those diagnosed. For example, some participants compared their experience to that of others’ experiences with dementia or other more intense forms of caregiving, noting their situation was “not as bad.” Nevertheless, if care partners at this stage of cognitive impairment already envision themselves as a more intensive type of caregiver in the future, and due to the increased risk of progression from MCI to dementia (Eshkoor et al., 2015), investing in support services to sustain the mental health and well-being of care partners during the MCI stage appears critical. Investing in such services must first be informed by addressing the current underutilization of caregiver support services that are already available.

### Paradoxical implications of caregiver identity

An overarching explanation for the underutilization of caregiver support services is that many individuals helping with care tasks do not identify as caregivers (Eifert et al., 2015). Nearly all of our participants (*n* = 17) performed care tasks, yet only three were clearly identifying as caregivers. Encouraging caregiver identity may improve uptake of support services (Andréasson et al., 2018), and may promote a sense of belonging to the broader caregiving community (O’Connor, 2007). Conversely, strongly encouraging care partners to identify might have the opposite effect, given the ambiguity and mild nature of MCI, which may complicate how care partners view themselves in relation to the person diagnosed (Gomersall et al., 2015). Pressuring individuals to adopt a caregiver identity may further promote caregiver need minimization as care partners work to protect the identity and dignity of those diagnosed (Moore & Gillespie, 2014). These potential consequences may be particularly true for MCI care partners, based on their interpretations of what it means to be a caregiver. For care partners who interpret caregiving more negatively (e.g., as a burden, power imbalance, and confusion), enforcing a caregiver label may elicit difficult emotions.

Alternatively, it may not be necessary, or even beneficial, to encourage a caregiver identity in MCI care partners. Instead, simply recognizing this lack of self-identification can inform how we develop and advocate for such supports. For example, public service initiatives and support groups might be encouraged to use neutral terms (e.g., family and friends of people diagnosed with MCI) to attract care partners. This recommendation is consistent with research that calls for outreach strategies to attract caregivers who do and do not self-identify, for caregiver support programs (Corden & Hirst, 2011; Funk, 2019). Ultimately, amid debates about whether it is advisable to promote caregiver identification, it is important to reflect on the complex and potentially paradoxical implications of the label itself for those supporting someone with MCI. What does remain clear from our findings, as well as the MCI literature (Dean et al., 2014; Morris et al., 2020; Paradise et al., 2015; Seeher et al., 2013), is that irrespective of how care partners identify, they are engaged in care tasks, and would likely benefit from support with navigating these changes and their new and evolving roles.

### Limitations and future directions

In addition to the strengths of this study, there are limitations of which to be mindful. The focus of this study was on participant experiences and interpretations; there is no expectation that the particular findings are generalizable. Nevertheless, we recruited a rather homogenous sample (e.g., most were white, middle SES, and all participants spoke fluent English) from a specific clinic in one geographic location. As such, we could not examine how diverse cultural frameworks may affect caregiver identity and service access. Neither did we explore identity development during the earliest stage of MCI that preceded participants’ pursuit of assessment and support services via the clinic. There is also the risk of selection bias as it is possible that the experiences of people who did not wish to participate in this study differ from those who agreed to participate. Additionally, although it was not an aim of this study to compare the identity development of different types of care partners (i.e., children, spouses, and friends), future research should explore whether caregiver identity emerges in similar or different ways depending on factors such as caregiver age, and the nature and intensity of the relationship with the care recipient. Last, due to the risk of increasing decline in persons with MCI (Petersen et al., 2014), future work should also explore how the themes identified in our study evolve to predict changes in caregiver identity development over time, as well as within different MCI types and stages of severity. Nonetheless, this study provides a nuanced conceptual contribution to the understanding of the unique and evolving caregiver identity development in family and friends of persons living with MCI.

## Supplemental Material

sj-pdf-1-dem-10.1177_1471301221994317 – Supplemental Material for Caregiver identity in care partners of persons living with mild cognitive impairment Supplemental Material, sj-pdf-1-dem-10.1177_1471301221994317 for Caregiver identity in care partners of persons living with mild cognitive impairment by Brooke E Beatie, Corey S Mackenzie, Laura Funk, Dylan Davidson, Lesley Koven and Kristin A Reynolds in Dementia
