# Supplementary material for: Caregiver identity in care partners of persons living with mild cognitive impairment
Source: Dementia (London). 2021 Feb 17;20(7):2323–39. doi: 10.1177/1471301221994317 (PMC8564256; doi:10.1177/1471301221994317)
Supplement: sj-pdf-1-dem-10.1177_1471301221994317 – Supplemental Material for Caregiver identity in care partners of persons living with mild cognitive impairment [file sj-pdf-1-dem-10.1177_1471301221994317.pdf]

Supplementary Material

Interview Guide

Preamble statement: Thanks for taking the time today to meet with me. The questions I will be asking you today are to help me understand the experiences of people like you who are here to support people with memory or thinking difficulties. Additionally, when individuals experience memory/thinking changes, it can present challenges for people in their life, so I'm also going to ask questions about how you are doing. Today, I'll be spending the next hour or so talking about this, feel free to talk as much as you like. I really just want to understand your experience the best way that I can. If there are some questions you don't feel comfortable answering, or if you would like to stop the interview at any time, that is ok, just let me know. Feel free to let me know if you have any questions at any time.

1. Can you start by telling me about your relationship with X? [*probe for how they identify their relationship; are they the primary caregiver, or do they refer to themselves as a son/wife/daughter etc.]* I'm interested to know what the relationship is, how long you've known X, how much time you spend with her/him, etc.

   a. How has their memory difficulties affected your relationship? *[some folks may answer this question with just tasks. If so, probe for how this have impacted other dimensions of their relationship. E.g., how you relate to each other, how you feel about him or her?]*

2. Tell me about the cognitive changes that X has been experiencing.

   a. How long has this been going on?

   b. What kind of changes have happened

   c. When did you notice something was wrong, etc.

3. Now that I have a good sense of your relationship, and of the reasons that brought you to ECCCOA with X, I would like you to tell me how your relationship has changed since X started experiencing memory problems.

   a. What was your relationship like before you noticed changes in X's memory/thinking? [probe for prior relationship quality vs now.

i. If no change: What do you think has contributed to this? Has anything not changed? *(try to get a picture of their daily life. The more changes the more likely their identity will change)*

ii. If changes: What kind of changes? What did these changes mean to you? How did feel about it, what do these changes look like? Did they change you?

4. It's often the case that the focus of attention is on people who are going through health challenges. What about you – have you noticed any changes in yourself since X started experiencing memory problems?

a. What about you has remained the same? [probe for societal, cultural, familial influences]

5. How have you been coping with these changes?

a. What do you do to manage these changes?

b. Have they been difficult?

i. Were there times when you've felt more distressed?

ii. What about times when you felt less distressed?

6. Have you had earlier life experiences that have helped prepare you to care for or support X in the way you do?

7. Have you heard of the term "caregiver"? – yes/no What is your understanding of the this word?

a. What does that term mean to you?

b. Do you think that applies to you in any way?

8. Has anyone used the term "caregiver" to describe you in relation to X before?

a. If yes:

i. When was the first time you heard it?

ii. How did you react?

b. If no:

i. What is your reaction to that term now? [probe to see if they may be avoiding this term/resisting the label]

ii. Do you think that you might consider yourself a caregiver to X at some point in the future?

iii. What would it mean if you were a caregiver?

9. Would you consider yourself a caregiver for X?

a. If yes:

i. How does it feel, when you think of yourself as a caregiver for X?

ii. How is it different from being a [daughter/etc]?

iii. When did you start to think of yourself in that way? [probe for previous history of caregiving, how did they internalize that role, how did other people treat them]

b. If no:

i. How do you primarily identify then in relation to this person?

ii. Can you talk about how you view the roles as different (or not)?

10. Is there anything else that you would like to say before we end the interview?
